# Are sleep disorders associated with increased mortality in asthma patients?

**DOI:** 10.1186/s12890-016-0313-2

**Published:** 2016-11-17

**Authors:** Kyu-Tae Han, Hong-Chul Bae, Sang Gyu Lee, Seung Ju Kim, Woorim Kim, Hyo Jung Lee, Yeong Jun Ju, Eun-Cheol Park

**Affiliations:** 1 Department of Public Health, Graduate School, Yonsei University, Seoul, Republic of Korea; 2 Institute of Health Services Research, Yonsei University College of Medicine, Seoul, Republic of Korea; 3 Office of Communication, Korea Centers for Disease Control and Prevention, Cheongju, Republic of Korea; 4 Department of Hospital Management, Graduate School of Public Health, Yonsei University, Seoul, Republic of Korea; 5 Department of Preventive Medicine, Yonsei University College of Medicine, Seoul, Republic of Korea

**Keywords:** Sleep disorders, Asthma, Mortality, Healthcare accessibility, Vulnerable population

## Abstract

**Background:**

South Korea has experienced problems regarding poor management of symptoms of asthma patients and remarkable increases in sleep disorders. However, few studies have investigated these issues. We examined the relationship between sleep disorders and mortality in asthma patients to suggest effective alternatives from a novel perspective.

**Methods:**

We used data from the National Health Insurance Service (NHIS) National Sample Cohort 2004–2013, which included medical claims filed for 186,491 patients who were newly diagnosed with asthma during the study period. We performed survival analyses using a Cox proportional hazards model with time-dependent covariates to examine the relationship between sleep disorders and mortality in asthma patients.

**Results:**

There were 5179 (2.78%) patients who died during the study period. Sleep disorders in patients previously diagnosed with asthma were associated with a higher risk of mortality (hazard ratio [HR]: 1.451, 95% confidence interval [CI]: 1.253–1.681). In addition, significant interaction was found between sleep disorders and Charlson comorbidity index.

**Conclusions:**

Our findings suggest that an increased prevalence of sleep disorders in asthma patients increases the risk of mortality. Considering the worsening status of asthma management and the rapid growth of sleep disorders in South Korea, clinicians and health policymakers should work to develop interventions to address these issues.

## Background

Asthma is a common chronic disease largely caused by genetic and environmental risk factors such as indoor and outdoor allergens, smoking, chemical irritants in the workplace, and air pollution. Its symptoms include recurring periods of wheezing, chest tightness, shortness of breath, and coughing [1]. In severe cases, asthma can lead to death. According to a World Health Organization (WHO) report, about 235 million people suffer from asthma worldwide, and it is especially severe for children in low- and middle-income countries [2]. Although asthma is not curable, optimal management can control symptoms and improve the quality of life of patients.

To determine the optimal management of asthma patients, numerous previous studies have been conducted, revealing many risk factors related to worsening status of asthma patients including clinical, socioeconomic, seasonal, and environmental factors [3–10]. Nevertheless, concerns remain regarding the management of worsening asthma in South Korea. According to the Organization for Economic Co-operation and Development (OECD) Health at a Glance 2015, asthma management in South Korea is worse than in other OECD countries; specifically, hospital admission rates for asthma patients are 98.5 per 100,000 people in South Korea compared to the average of 43.8 per 100,000 people in other OECD countries [11]. To solve the problems related to inappropriate management, the Health Insurance Review & Assessment service (HIRA) introduced a quality evaluation for asthma in 2013. However, it is also essential to assess problems related the poor level of asthma management from alternative perspectives. Here, we focus on the link between outcomes of asthma patients and sleep disorders. Sleep is intimately related to many aspects of an individual’s life, and sleep disorders may contribute to both physical and mental problems. According to previous studies, sleep disorders and problems such as low quality of sleep or short sleeping time could result in deteriorating conditions in patients with chronic diseases, including asthma, and in severe cases, sleep disorders were even associated with mortality in patients [12–14].

In South Korea, sleep issues have become increasingly common. According to National Health Insurance Service (NHIS) reports, health resource utilization due to organic sleep disorders (International Classification of Diseases [ICD]-10: G47) has gradually increased in South Korea, with the number of inpatients and outpatients growing from 358,062 in 2012 to 380,876 in 2013 and 414,524 in 2014 [15]. Now, it has become a major concern that needs to be solved and has even gained the media spotlight recently. Despite these remarkable increases over the last several years, few studies have investigated the negative impact of sleep disorders in South Korea. In this study, we analyzed the association between sleep disorders and mortality as the outcome variable among patients who were diagnosed with asthma, thereby considering the poor quality of asthma patient management and considerable increases in sleep disorders in South Korea. Based on this study, we expected to be able to develop alternatives for the optimal management of asthma in South Korea.

## Methods

### Study population

The data used in this study were obtained from the NHIS National Sample Cohort 2002–2013 that was released in 2014. They comprised a nationally representative random sample of 1,025,340 individuals, approximately 2.2% of the entire NHIS population in 2002. The data were produced by the NHIS using a systematic sampling method to generate a representative sample of 46,605,433 Korean residents recorded in 2002. The database included all medical claims filed from January 2002 to December 2013. To investigate the association between sleep disorders and mortality in patients with diagnosed asthma, we included only patients who were newly diagnosed with asthma (ICD-10: J45) during outpatient care after 2004. We then excluded patients diagnosed with a sleep disorder (ICD-10: G47) prior to the diagnosis of asthma to limit our investigation to the effect of sleep disorders on patients who had already experienced asthma. Ultimately, the data used in this study were from 186,491 patients newly diagnosed with asthma from 2004–2013. Regional characteristics were determined from the “e-provincial indicators” published by Statistics Korea, which contained the regional demographic structures for all 253 basic administrative *si-gun-gu* (city-county-ward) districts of South Korea. We classified the data based on the *si-gun-gu* information to take into account the regional characteristics of the community in which each patient resided (Statistics Korea) [16].

### Variables

The outcome variable was mortality in patients diagnosed with asthma (ICD-10: J45). Mortality was defined as all types of death in patients with asthma during the study period. We identified the date of each patient’s first hospital visit through either outpatient care or admission during the study period, and we followed each patient after that date.

The primary independent variable in relation to the mortality of patients with asthma was whether they were diagnosed with an organic sleep disorder during follow-up (ICD-10: G47). We adjusted both patient and regional variables when analyzing the effect of sleep disorders on mortality in patients with asthma. The patient variables included in the analyses were sex, age, income, insurance coverage type, year of first asthma diagnosis, disability, Charlson comorbidity index score, and average annual pharmaceutical expenditures for asthma. Age was divided into 10-year groups (≤19, 20–29, 30–39, 40–49, 50–59, 60–69, and ≥70 years) to reflect any differences in the effects of sleep disorders on mortality among patients with asthma by age group. Income level was categorized into deciles based on mean household income as follows: ≤10%, 11%–20%, 21%–30%, 31%–40%, 41%–50%, 51%–60%, 61%–70%, 71%–80%, 81%–90%, and ≥91%. The types of insurance coverage were categorized as medical aid, National Health Insurance (NHI) employee insurance, or NHI self-employed insurance based on the NHI criteria. Those with NHI employee insurance included workers and employers in all workplaces, public officials, private school employees, continuously insured persons, and daily paid workers at construction sites. Beneficiaries of NHI employee insurance included spouses, descendants, siblings, and parents. People with NHI employee insurance paid a regular portion (about 7%) of their average salary in contribution payments, and the rates changed every year. The NHI self-employed insurance category included people who did not fall into the above-described group. Their contribution amount was set by taking into account their income, property, living standard, and rate of participation in economic activities. Medical aid beneficiaries were defined as patients with an income below the government-defined poverty level or a disability who were provided with free inpatient and outpatient care paid with government funds. Therefore, the type of insurance coverage represented each patient’s socioeconomic status [17, 18]. The first diagnosis for asthma was defined as the year that each patient was initially diagnosed and was included to reflect the duration of illness. Disability was classified as none, mild, and severe to reflect the effect of disability on worsening health outcomes in patients with asthma. The Charlson comorbidity index was calculated by weighting and scoring for comorbid conditions, with additional points added to consider comorbidities that could affect health outcomes of patients with asthma. Average annual pharmaceutical expenditures for asthma were calculated as the average of the sum of pharmaceutical expenditures every year after asthma diagnosis (ICD-10: J45). This could indirectly reflect the level of treatment and asthma severity.

The regional variables were region, number of medical centers per 1000 residents, population size, proportion of seniors, and financial independence rate of the local government. The region types were metropolitan and other. Population size was defined as the total number of residents in each community, and the proportion of seniors was the number of elderly individuals among the entire community. The financial independence rate of the local government was an index of the finance utilization capacity of a local government with independent discretionary power. This indicator was calculated as follows: (local taxes + non-tax revenue)/local government budgets × 100.

### Statistical analysis

We first examined the frequencies and percentages of each categorical variable at each patient’s baseline and performed *χ* ^2^ tests to examine the distribution for mortality according to each variable. We examined the mean and standard deviation of each continuous variable at baseline and performed *t*-tests or Wilcoxon-Mann–Whitney tests for each variable by mortality during the study period. Analyses were performed for both patient- and regional-level variables. Kaplan-Meier survival curves and log-rank tests were used to compare survival rates between groups. We performed survival analyses using a Cox proportional hazards model with time-dependent covariates to examine factors associated with mortality [19]. Subgroup analyses by age group, insurance coverage type, average annual pharmaceutical expenditures for asthma, region, CCI, and number of medical centers were used to investigate the relationship between sleep disorders and mortality in patients with asthma. All statistical analyses were performed using SAS statistical software version 9.2 (Cary, NC).

## Results

The data used in the analysis were collected from 186,491 patients with asthma. Table 1 shows their general characteristics at baseline (at asthma diagnosis) and the descriptive statistics for mortality. There were 5179 patients (2.78%) who died during the study period (major causes of death = lung cancer: 10.5%, senility: 6.1%, COPD: 4.5%, myocardial infarction: 4.4%, and stomach cancer: 3.7% among total deaths). The average follow-up period was 62.17 months. Patient with sleep disorders more frequently appeared in the died criteria than the survived criteria. Those who were medical aid beneficiaries also had a higher risk, as did patients with longer illness durations, severe disabilities, more comorbidities, and higher pharmaceutical expenditures. With regard to regional variables, patients in non-metropolitan regions had higher mortality.

**Table 1:** General characteristics of the study population

Variable	Total		Died		Survived		*P*-value
	n/Mean	%/SD	n/Mean	%/SD	n/Mean	%/SD	
Sleep disorder							
Yes	4,565	2.45	320	6.18	4,245	2.34	<0.0001
No	181,926	97.55	4,859	93.82	177,067	97.66	
Sex							
Male	83,801	44.94	2,819	54.43	80,982	44.66	<0.0001
Female	102,690	55.06	2,360	45.57	100,330	55.34	
Age (years)							
≤19	80,859	43.36	62	1.20	80,797	44.56	<0.0001
20–29	13,342	7.15	26	0.50	13,316	7.34	
30–39	21,861	11.72	89	1.72	21,772	12.01	
40–49	20,849	11.18	213	4.11	20,636	11.38	
50–59	18,911	10.14	449	8.67	18,462	10.18	
60–69	16,170	8.67	1,047	20.22	15,123	8.34	
≥70	14,499	7.77	3,293	63.58	11,206	6.18	
Income (percentile)							
≤10 (low)	14,779	7.92	816	15.76	13,963	7.70	<0.0001
11–20	10,388	5.57	330	6.37	10,058	5.55	
21–30	11,584	6.21	370	7.14	11,214	6.18	
31–40	13,884	7.44	405	7.82	13,479	7.43	
41–50	16,266	8.72	370	7.14	15,896	8.77	
51–60	19,443	10.43	421	8.13	19,022	10.49	
61–70	22,858	12.26	440	8.50	22,418	12.36	
71–80	26,097	13.99	542	10.47	25,555	14.09	
81–90	26,628	14.28	716	13.83	25,912	14.29	
≥91 (high)	24,564	13.17	769	14.85	23,795	13.12	
Insurance coverage							
Medical aid	3,934	2.11	269	5.19	3,665	2.02	<0.0001
NHI, self-employed insured	63,816	34.22	2,044	39.47	61,772	34.07	
NHI, employee insured	118,741	63.67	2,866	55.34	115,875	63.91	
Year of first diagnosis of asthma							
2004	23,728	12.72	1,116	21.55	22,612	12.47	<0.0001
2005	23,724	12.72	920	17.76	22,804	12.58	
2006	21,994	11.79	709	13.69	21,285	11.74	
2007	19,922	10.68	656	12.67	19,266	10.63	
2008	19,109	10.25	597	11.53	18,512	10.21	
2009	18,064	9.69	455	8.79	17,609	9.71	
2010	16,369	8.78	267	5.16	16,102	8.88	
2011	16,521	8.86	268	5.17	16,253	8.96	
2012	14,947	8.01	149	2.88	14,798	8.16	
2013	12,113	6.50	42	0.81	12,071	6.66	
Disability							
None	179,110	96.04	4,304	83.10	174,806	96.41	<0.0001
Mild	5,874	3.15	581	11.22	5,293	2.92	
Severe	1,507	0.81	294	5.68	1,213	0.67	
Charlson comorbidity index							
≤1	126,882	68.04	328	6.33	126,554	69.80	<0.0001
2	24,752	13.27	405	7.82	24,347	13.43	
3	16,685	8.95	890	17.18	15,795	8.71	
≥4	18,172	9.74	3,556	68.66	14,616	8.06	
Average pharmaceutical expenditures for asthma in each year (KRW)	24,773.30	±63,689.71	51,765.29	±111,475.00	24,002.30	±61,611.30	<0.0001
Region							
Metropolitan	83,723	44.89	2,001	38.64	81,722	45.07	<0.0001
Other	102,768	55.11	3,178	61.36	99,590	54.93	
Number of medical centers per 1000 residents	9.17	±4.39	9.89	±5.11	9.15	±4.37	<0.0001
Population size	420,845.64	±259,860.18	351,277.38	±259,532.33	422,832.79	±259,596.53	<0.0001
Proportion of seniors (%)	12.09	±4.94	14.16	±6.42	12.03	±4.87	<0.0001
Financial independence rate of local government (%)	59.06	±21.79	53.6	±22.60	59.22	±21.74	<0.0001
Average follow-up (months)	62.17	±34.26	38.43	±28.57	62.85	±34.17	<0.0001

*SD* standard deviation; *NHI* National Health Insurance; *KRW* Korean Won

**Fig. 1:**
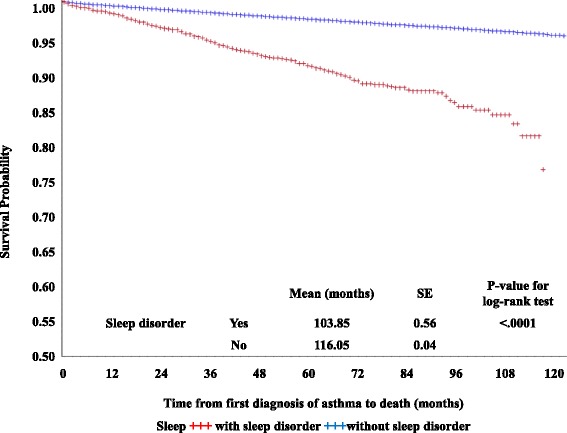
Kaplan-Meier survival curves and log-rank test results comparing survival rates between patients with asthma, with (+++, dotted line) or without a sleep disorder (solid line)

Figure 1 shows Kaplan-Meier survival curves and log-rank test results of the study population. The average period from first diagnosis to death was shorter in patients with sleep disorders (mean: 103.85 months, standard error [SE]: 0.56) than in those without sleep disorders (mean: 116.05, SE: 0.04; *p* < 0.0001).

Table 2 shows the results of the survival analysis using a Cox proportional hazard model with time-dependent covariates to investigate the relationship between sleep disorders and mortality in patients with asthma. Those with sleep disorders had a greater risk of mortality (with sleep disorder, hazard ratio [HR]: 1.451, 95% confidence interval [CI]: 1.253–1.681; reference: without sleep disorder). Males and older patients were at greater risk, as were asthma patients with lower incomes and those who received medical aid (medical aid, HR: 1.546, 95% CI: 1.317–1.814; NHI self-employed insurance, HR: 1.126, 95% CI: 1.061–1.195; reference: NHI employee insurance). For regional variables, patients in metropolitan regions were at lower risk, and higher financial independence of the local government was inversely associated with mortality risk.

**Table 2:** Cox proportional hazard model for association between sleep disorders and mortality

	HR	95% CI		*P*-value
		Lower	Upper	
Sleep disorder				
Yes	1.451	1.253	1.681	<0.0001
No	1.000	-	-	-
Sex				
Male	2.002	1.893	2.117	<0.0001
Female	1.000	-	-	-
Age (years)				
≤19	1.000	-	-	-
20–29	2.865	1.809	4.540	<0.0001
30–39	5.823	4.192	8.088	<0.0001
40–49	14.144	10.603	18.866	<0.0001
50–59	24.786	17.431	35.243	<0.0001
60–69	57.397	39.115	84.224	<0.0001
≥70	207.336	138.828	309.651	<0.0001
Income (percentile)				
≤10 (low)	1.375	1.228	1.540	<0.0001
11–20	1.390	1.219	1.585	<0.0001
21–30	1.439	1.267	1.634	<0.0001
31–40	1.476	1.306	1.668	<0.0001
41–50	1.289	1.136	1.461	<0.0001
51–60	1.337	1.185	1.507	<0.0001
61–70	1.240	1.101	1.396	0.0004
71–80	1.227	1.098	1.371	0.0003
81–90	1.102	0.994	1.221	0.0651
≥91 (high)	1.000	-	-	-
Insurance coverage				
Medical aid	1.546	1.317	1.814	<0.0001
NHI, self-employed insured	1.126	1.061	1.195	<0.0001
NHI, employee insured	1.000	-	-	-
Year of asthma diagnosis				
2004	1.000	-	-	-
2005	0.884	0.808	0.967	0.0069
2006	0.765	0.694	0.844	<0.0001
2007	0.859	0.776	0.951	0.0035
2008	0.770	0.692	0.858	<0.0001
2009	0.784	0.697	0.882	<0.0001
2010	0.637	0.552	0.734	<0.0001
2011	0.603	0.518	0.702	<0.0001
2012	0.603	0.501	0.727	<0.0001
2013	0.671	0.487	0.925	0.0148
Disability				
None	1.000	-	-	-
Mild	1.214	1.113	1.326	<0.0001
Severe	2.909	2.580	3.280	<0.0001
Charlson comorbidity index				
≤1	1.000	-	-	-
2	1.437	1.122	1.840	0.0041
3	1.568	1.169	2.105	0.0027
≥4	2.019	1.467	2.779	<0.0001
Average annual pharmaceutical expenditures for asthma (per 10,000 KRW increase)	1.003	1.000	1.005	0.018
Region				
Metropolitan	0.965	0.897	1.039	0.3419
Other	1.000	-	-	-
*Number of medical centers per 1000 residents (per 5 increase)*	1.016	0.987	1.047	0.2789
*Population size (per 100,000 increase in population)*	0.977	0.962	0.992	0.0029
*Proportion of seniors (per 10% increase)*	0.940	0.883	1.000	0.0502
*Financial independence rate of local government (per 10% increase)*	0.978	0.961	0.996	0.0166

*CI* confidence interval; *HR* hazard ratio; *NHI* National Health Insurance; *KRW* Korean Won

We performed interaction tests to further examine potential effect modification of the associations between sleep disorders and mortality by age, insurance coverage, pharmaceutical expenditures, region, number of medical centers per 1000 residents and the CCI. Except for CCI, the interaction terms between sleep disorders and the mentioned variables were not statistically significant. Nevertheless, a trend in the magnitude of the stratified HR was observed for age and insurance coverage. For age, the highest HR were observed among younger age groups. For insurance coverage, higher HR were observed for NHI self-employed insurance than for NHI employee insurance. In addition, the association between sleep disorders and mortality was stronger in non-metropolitan regions compared with metropolitan areas, and among individuals with lower number of medical centers per 1000 residents. For CCI, the interaction term between sleep disorders and CCI was statistically significant. And, higher HR were observed among higher CCI group (Table 3).

**Table 3:** Subgroup analysis using Cox proportional hazard model by age, insurance coverage, average pharmaceutical expenditures, region, and number of medical centers

	HR	95% CI		*P*-value
		Lower	Upper	
Age (years)				
≤29	7.351	1.735	31.139	0.0068
30–59	1.415	0.865	2.313	0.1665
≥60	1.301	1.097	1.543	0.0025
Insurance coverage				
Medical aid	0.690	0.316	1.508	0.3518
NHI, self-employed insured	1.647	1.305	2.078	<0.0001
NHI, employee insured	1.327	1.083	1.626	0.0063
Average annual pharmaceutical expenditures for asthma				
Below median	1.447	1.116	1.876	0.0052
Above median	1.466	1.229	1.751	<0.0001
Region				
Metropolitan	1.370	1.087	1.727	0.0078
Other	1.508	1.248	1.825	<0.0001
Number of medical centers per 1000 residents				
Below median	1.609	1.302	1.988	<0.0001
Above median	1.323	1.079	1.622	0.0071
Charlson comorbidity index				
≤2	1.359	1.148	1.610	0.0004
≥3	1.553	1.020	2.364	0.0401

*CI* confidence interval; *HR* hazard ratio; *NHI* National Health Insurance. The results of the subgroup analysis to investigate differences in the association between sleep disorders and mortality according to age, insurance coverage, average annual pharmaceutical expenditures for asthma, region, number of medical centers, and Charlson comorbidity index. The *P*-value in type 3 tests for models with interactions between sleep disorders and subgroup variables were as follows: model with interaction between age and sleep disorders, *P*-value in type 3 test = 0.2249; model with interaction between insurance coverage and sleep disorders, *P*-value in type 3 test = 0.1742; model with interaction between average annual pharmaceutical expenditures for asthma and sleep disorders, *P*-value in type 3 test = 0.4199; model with interaction between region and sleep disorders, *P*-value in type 3 test = 0.3216; model with interaction between number of medical centers and sleep disorders, *P*-value in type 3 test = 0.6448; model with interaction between Charlson comorbidity index and sleep disorders, *P*-value in type 3 test = 0.0427

## Discussion

In the past several decades, South Korea has experienced rapid social and economic growth. The health status of South Koreans has also improved, and their lifespan has increased accordingly. Although burdens of illness due to acute diseases have decreased, chronic conditions now present new challenges [20]. Many researchers and healthcare professionals have made efforts to control such health problems including cancer, cardiovascular diseases, and cerebrovascular diseases, although certain issues remain. Specifically, relatively mild chronic diseases are not well managed, including asthma. In this area, South Korea lags behind other OECD countries [11]. According to health professionals, the difficulties of asthma management in South Korea are related to shortcomings in primary care, and they have suggested that continuity of care for asthma patients through primary care could improve health outcomes in this population. However, innovative changes in South Korean primary care are difficult to implement [21]. We focused on the relationship between sleep disorders and mortality in asthma patients to determine if addressing this issue could help this patient population.

Our findings suggest that the presence of sleep disorders in patients previously diagnosed with asthma is associated with an increased mortality risk. Previous studies have reported that sleep disorders affect overall quality of life and can deteriorate patients physically and psychologically [22–25]. Given the worsening status of asthma management and increasing numbers of patients diagnosed with sleep disorders, we specifically focused on the relationship between sleep disorders and mortality in patients with asthma. In South Korea, patients have been able to receive healthcare with 20%–30% copayment since the introduction of NHI in 1989 [26]. However, patients who visit medical institutions due to sleep disorders only receive health insurance coverage for sleep apnea based on the criterion of the respiratory distress index. This suggests that certain patients may not have received appropriate treatment. Therefore, it may be appropriate to revise the insurance coverage criteria to reduce avoidable death in patients with asthma. Additionally, improving the accessibility of healthcare by reducing copayments via insurance policy changes may prevent the worsening status of asthma patients with sleep disorders, even if there is no direct impact on mortality. Furthermore, we expect that such an impact may be helpful in patients with asthma as well as in patients with other chronic diseases, from a health policy perspective.

Subgroup analyses revealed several interesting results. The increased mortality in patients with asthma and sleep disorders was greater for younger patients, those with NHI self-employed insurance, those in non-metropolitan areas, and those in regions with low healthcare accessibility. First, younger patients had more negative outcomes, which had been previously reported [27]. This suggests that careful management for younger asthma patients could prevent their condition from worsening. Second, patients with sleep disorders who had NHI self-employed insurance had a higher mortality rate. This indicates that the evaluation criteria for beneficiaries of health insurance could benefit from revision. In South Korea, the government medical aid program is for patients with an income below the government-defined poverty level. However, the evaluation for patients who receive NHI self-employed insurance could have overestimated their socioeconomic level compared to those with NHI employee insurance due to evaluation methods. It is therefore possible that they could have more limited access to healthcare and might not receive appropriate treatment. Based on these results, health policymakers must reconsider the evaluation methods for health insurance beneficiaries [28]. Also, differences in regional characteristics could affect patient outcomes. This is another issue that could be addressed with government interventions. Finally, the significant interaction between sleep disorders and the CCI was observed in this study. As already mentioned, CCI was usually used to consider comorbidities that could affect health outcomes of patients. Thus, the presence of interaction means that the occurrence of sleep disorder in asthma patient with more comorbidities could affect to more worsening their status, and could not well manage their condition than patient with less comorbidities [29]. Therefore, healthcare professionals were needed to carefully provide care for patient considering their comorbidity condition.

Our study had several strengths compared to previous publications. First, we used national sampling cohort data to assess the relationship between sleep disorders and mortality risk in patients with asthma. Such data are particularly helpful for establishing evidence-based policies to effectively manage asthma patients. Next, to the best of our knowledge, this study is the first study to investigate the impact of sleep disorders on the mortality risk of asthma patients in South Korea. Other previous reports described the negative effects of sleep disorders and relationships between sleep disorders and health outcomes. However, few studies have regarded the relationship between sleep disorders and mortality in patients with asthma in South Korea. Thus, our findings could provide useful evidence for the development of policies or programs to better manage patients with asthma and sleep disorders. Finally, we made a concerted effort to reduce the limitations of secondary data by adjusting covariates for patients’ clinical and regional characteristics. We included the year of asthma diagnosis, disability, Charlson comorbidity index score, and average annual pharmaceutical expenditures due to asthma to reflect disease severity and included regional characteristics to consider differences in medical accessibility by area.

Our study also had several limitations. Although several factors that affect mortality were included in this study, more detailed information such as socioeconomic factors was not available [30]. Given that the average period from the first diagnosis for asthma to death was about 9 years, there were several potential confounders related to managing asthma, such as detailed severity indicators and smoking status, which could have influenced the health outcomes of patients with asthma. Unfortunately, we could not assess such confounders in this study due to data limitations, as the data used in this study were claim data that were collected to provide payment for both patients and providers based on medical utilization. Second, we did not have detailed information regarding patient income level, as the data were collected based on NHI criteria for the overall economic status of a population; detailed income described in monetary units was not included. Third, in investigating the association between the occurrences of sleep disorders and morality as an outcome variable, we only focused on patients with asthma rather than the general population. Therefore, there were several concerns regarding the external validity of the whole population in this study. Finally, the occurrences of sleep disorders may have decreased as the status of patients with asthma worsened; thus, they may not have been a direct risk factor for death. However, we only used all-cause mortality as an outcome variable in this study rather than measuring symptom indicators such as pulmonary function test results. Therefore, we were unable to consider the other changes by occurrences of sleep disorders among patients with asthma in this study, and this may have caused over- or underestimation of the impact of sleep disorders. In addition, although the data included detailed causes of death, the sample size was too small to analyze the covariates used in this study (e.g., respiratory-specific mortality: 0.34%).

Despite these limitations, our findings suggest that sleep disorders in patients previously diagnosed with asthma are negatively associated with their health status, and in particular, mortality. Although further studies using more detailed data will be needed, these results underscore the need for health policymakers and healthcare professionals to identify effective ways to reduce the prevalence of sleep disorders in South Korea.

## Conclusion

Sleep disorders in asthma patients are associated with higher mortality. Considering the worsening status of asthma management and the rapid growth of sleep disorders in South Korea, health policymakers should consider developing effective interventions based on our findings.

## Abbreviation

NHIS: National Health Insurance Service
HR: Hazard ratio
CI: Confidence interval
WHO: World Health Organization
OECD: Organization for Economic Co-operation and Development
ICD: International Classification of Diseases
NHI: National Health Insurance
SE: Standard error
